# Exploring the context of diacidic motif DE as a signal for unconventional protein secretion in eukaryotic proteins

**DOI:** 10.12688/wellcomeopenres.14914.1

**Published:** 2018-11-19

**Authors:** Sreedevi Padmanabhan, Malay Ranjan Biswal, Ravi Manjithaya, Meher K. Prakash

**Affiliations:** 1 Autophagy Laboratory, Molecular Biology and Genetics Unit, Jawaharlal Nehru Centre For Advanced Scientific Research, Bangalore, Karnataka, 560064, India; 2 Computational Biophysics Group, Theoretical Sciences Unit, Jawaharlal Nehru Centre For Advanced Scientific Research, Bangalore, Karnataka, 560064, India

**Keywords:** Diacidic motif, Unconventional protein secretion, LIR, charge, order, hydrophobicity

## Abstract

Unconventional protein secretion (UPS) is an important phenomenon with fundamental implications to cargo export. How eukaryotic proteins transported by UPS are recognized without a conventional signal peptide has been an open question. It was recently observed that a diacidic amino acid motif (ASP-GLU or DE) is necessary for the secretion of superoxide dismutase 1 (SOD1) from yeast under nutrient starvation. Taking cue from this discovery, we explore the hypothesis of whether the diacidic motif DE, which can occur fairly ubiquitously, along with its context, can be a generic signal for unconventional secretion of proteins. Four different contexts were evaluated: a physical context encompassing the structural order and charge signature in the neighbourhood of DE, two signalling contexts reflecting the presence of either a phosphorylatable amino acid (‘X’ in XDE, DXE, DEX) or an LC3 interacting region (LIR) which can trigger autophagy and a co-evolutionary constraint relative to other amino acids in the protein interpreted by examining sequences across different species. Among the 100 proteins we curated from different physiological or pathological conditions, we observe a pattern in the unconventional secretion of heat shock proteins in the cancer secretome, where DE in an ordered structural region has higher odds of being a UPS signal.

## Introduction

Proteins need to be secreted outside the cell for several physiologically important reasons ^1– 3^. In eukaryotes, proteins with an N-terminal signal peptide are known to get conventionally secreted in a vesicular mode through the endoplasmic reticulum (ER) – Golgi secretory pathway (ER-Golgi-secretory vesicles) ^4, 5^. However, interestingly, many proteins which lack in such well-defined signal peptides are also secreted, mostly under cellular stress ^6^. This important class of unconventional protein secretion (UPS) reflects the cellular response to stressors such as inflammation, nutrient stress, ER stress, mechanical stress, etc., and continues to grow in relevance as many such instances are detected in disease associated with dysfunctional autophagy such as neurodegeneration ^7^. Secretion of leaderless proteins (without signal peptides) is intriguing, and this non-canonical export mechanism raises several mechanistic questions on their presumably unique secretory pathways ^6^. While at least four different UPS mechanisms have been identified so far ^6, 8^, even simpler and fundamental questions on how these leaderless proteins are identified remain open.

Conventional signal peptides are 15–50 amino acid tags (“zip code”) at the N-terminus of the proteins ^4, 5, 9^. These signal peptides have a characteristic tripartite structure – positively charged N-region, a hydrophobic H-region, and a polar C-region; which makes it easier for the export machinery in the cells, as well as for the computational models, to sort the secretory from the non-secretory proteins. Several predictive models for identifying these signals have been developed ^10, 11^. On the other hand, identifying unconventionally secreted proteins has not been equally intuitive, as they lack in the pattern of canonical leader sequence ^4, 12^. Modelling attempts to predict the unconventional secretion have had limited success so far in deciphering the signatures of non-canonical secretion ^13^. Using artificial neural networks, which work by recognizing implicit patterns in protein sequences, unconventional secretory proteins were categorized by properties such as amino acid composition, secondary structure, and disordered regions, but do not explicitly reveal the patterns among the unconventional secretory proteins ^14, 15^. A rational basis for how non-classically secreted proteins are identified has been missing thus far.

In a recent landmark work, a motif which drives a protein through the non-canonical secretion pathway was identified for the first time. By systematically comparing the superoxide dismutase 1 (SOD1) from human and mouse cells with their extracellular SOD1 counterparts from human, mouse and yeast cells, a diacidic motif (Asp-Glu or DE) had been identified to be responsible for its unconventional secretion. Of the 6 amino acid insertion in SOD1, compared to its extracellular homologs, UPS was found to be sensitive to the mutation of two amino acids aspartate (D) and glutamate (E) ^16^. As a first and concrete observation, this finding raises the possibility that DE could be a generic UPS signal motif. However, as the short two amino acid length motif could occur in most proteins, in this study, we nucleate a minimal but rational hypothesis for the role of DE and its structural and charge context to act as a trigger for UPS.

## Methods

### Data curation

All the proteins used in this analysis are eukaryotic proteins. Since the hypothesis was centred on the contexts in which DE acts as a UPS trigger, any protein which had no DE motif was discarded from the analysis. The proteins used in the analysis were chosen from four different groups:- Group 1: Since it was recently demonstrated that the presence of DE in UPS of SOD1 ^16^ (cargo involved in neurodegenerative disease, Amyotrophic lateral sclerosis (ALS), we intended to check the same in all other neurodegeneration causing aggregates such as α-synuclein, β-amyloid, TDP-43, Tau followed by Group 2 that encompasses all the known UPS cargoes known to date. All these Group 2 secretory proteins were chosen from Keulers *et al.* ^17^, Group 3 were chosen from the dataset of heat shock proteins that are reported to be secreted in an unconventional manner in cancer ^18^ and the proteins from the breast cancer secretome where UPS is the major contributor ^19^. Group 4: While the list of non-secretory proteins could be very large, we did not add any more of them than the number of secretory proteins to keep the data set groups unbiased. Non–secretory proteins (cytoplasmic), were chosen from the Human Protein Atlas. The complete list of proteins used in the analysis is tabulated in Dataset 1 ^20^. The final curated set included 100 different eukaryotic proteins, 57 secretory and 43 non-secretory, whose DE sequence details, and the nature of protein are mentioned in Supplementary Table 1 ( Supplementary File 1) and Dataset 1 ^20^.

### Variables describing the physical context

**Figure 1.**
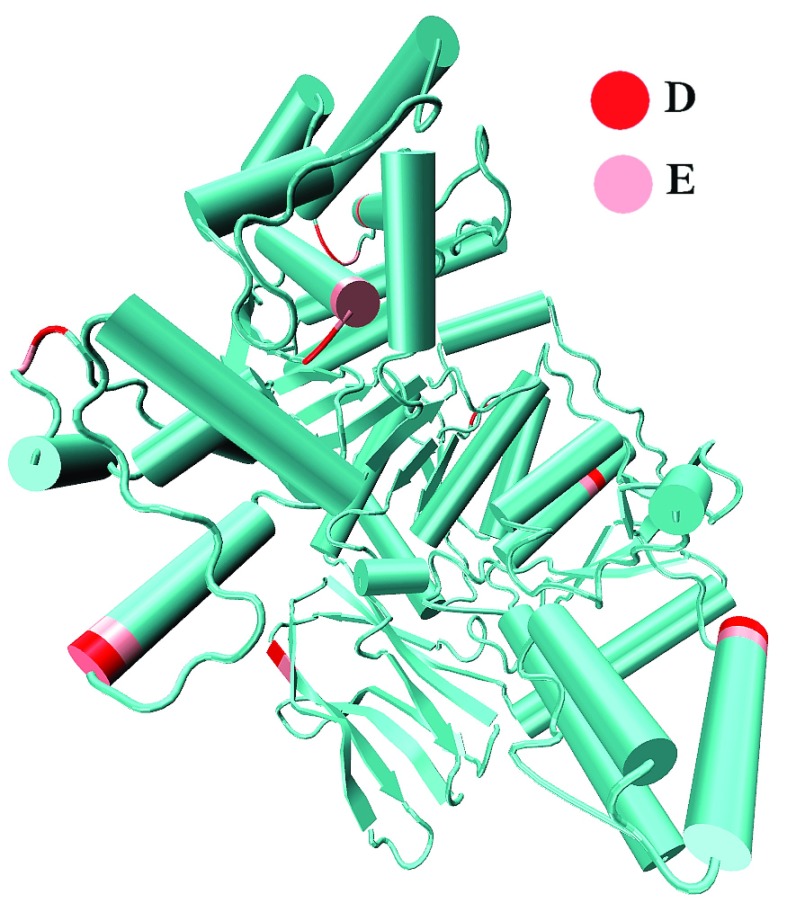
Illustration of the definition of structural order in NADP Isocitrate dehydrogenase (PDB:2B0T). The protein had multiple DE motifs, in ordered, loop and border regions.

 For each of the selected proteins, three parameters defining the context in which DE occurs in a protein were defined: (1) Hydrophobicity (H) of the local region, by adding the hydrophobicity score ^21^ of 3 amino acids on either side of the DE motif (2) Charge (C) of the local region, by summing the charge of 3 amino acids flanking on either side of DE and (3) structural order – the local structure around DE was initially categorically classified as ordered (O) if DE is in α- helix or β- sheet, disordered (D) if DE is in a loop or disordered region (as illustrated in Figure 1). If DE occurred in the border (B) of structured/unstructured regions, given the limited sample size of the data, we considered the option of regrouping B either as O or as D which is discussed below along with other cases.

### Most relevant DE motif

For every protein used in the analysis, we characterized all DE motifs by their physical descriptors (H,C,O/D/B). DE motif occurs in several places in many protein, and we wanted to identify the most important DE motif for UPS signalling using the analysis of the curated data. To identify the most relevant DE motif, we generated 16 possible hypotheses listed as cases in Supplementary Table 1A. The analyses were performed on all 16 data sets thus created.

### Multivariate binary logistic regression

For each of the 16 hypotheses, different DE motifs were considered to be important, and thus 16 different input files were prepared. Using these different data sets, and the binary prediction (secreted/not-secreted), we performed 16 independent binary logistic regression calculations using scikit-learn module (version 0.19.1) in Python (version 2.7) ^22^. Significance quantified by p-values were obtained from these Python analyses. The choice of the hypothesis comes *a posteriori*, comparing the quality of multivariate binary logistic regression model for the prediction of secretion ( Supplementary Tables 1B, Supplementary File 1).

### LIR motif analysis

We predicted the occurrence of microtubule-associated protein, LC3 interacting motifs in the proteins using the iLIR database ^23^. The details of the LIR motifs in the proteins analysed are tabulated in Dataset 1 ^20^.

### Co-evolution analysis

Homologous sequences and their multiple sequence alignments (MSA) for SOD1 and Acb1 proteins were obtained from the Pfam website using their identities Pf00080 and Pf00887 respectively. From the 3904 and 3489 sequences downloaded for the homologs of SOD1 and Acb1, we used a maximum gap frequency of 0.2, and used the selected sequences for co-evolutionary analyses. A consensus sequence was created by choosing the most common amino acid in each position. The entire MSA was then binarized, substituting 1 if the amino acid in a certain position is the same as the one in the consensus sequence, and 0 otherwise. A co-evolution matrix was created with a home-written Python code, following the protocol used in Statistical Coupling Analysis (SCA) ^24^. The pairwise co-evolution possibility was used to compare the co-evolutionary couplings different DE motifs have.

## Results

### Physical nature of the DE neighbourhood as a context

**Figure 2.**
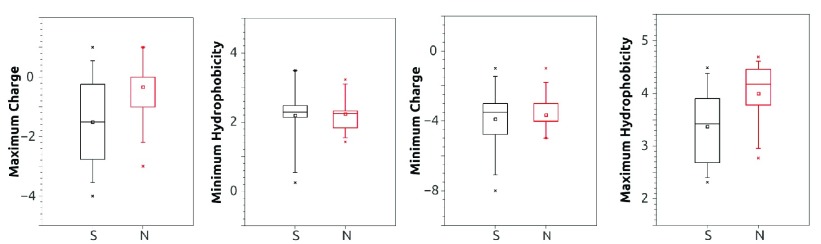
Dependence of secretory nature on the local properties of DE motif in cancer protein data (Groups 3B, 3C in Dataset 1 ^20^) on the extreme values of the descriptive parameters. ( **A**) Maximum charge ( **B**) minimum hydrophobicity ( **C**) minimum charge ( **D**) maximum hydrophobicity of the neighbouring amino acids were calculated for all the occurrences of the DE motifs, in each of the proteins appearing in the cancer secretome data.

***Dependency on single variable.*** Before building a multivariate predictive model, the curated data was classified as either secretory (UPS) or non-secretory, and their relation to each of the individually chosen variables describing the structural context of where DE occurs in the protein was examined. The secretory nature has a dependence, although not very strong, on all three physical context variables hydrophobicity (H) , charge (C), and (dis)order D/O that we calculated based on the protein sequence and structure data (see Methods). The most significant difference between the physical variables describing secreted and unsecreted proteins was observed in our analysis of the data from the cancer secretome ( Figure 2). Similar analysis for the other sets are represented in Supplementary Figure 1 ( Supplementary File 1).

***Multivariate regression.*** Since the three physical parameters showed a relation to the secretory nature, we combined all of them for a multivariate regression model in a binary logistic regression model to predict the odds of secretion. However, there are several DE motifs in most of the proteins, and we developed several hypotheses on how to identify the specific DE which may act as a putative UPS signal. These hypotheses were characterized by the choice of the extremes in the physical parameters used for describing each DE motif. An example of one such hypothesis is a composite assumption that among all the DEs occurring in the protein, DE in the ordered region is more likely to serve as a UPS signal, especially when it is flanked by amino acids with the lowest charge. For the simplicity of the analysis, the border region may be classified as disordered region, making the complete hypothesis as in case 9 in Supplementary Table 1A ( Supplementary File 1). With 16 different hypotheses ( Supplementary Table 1A, Supplementary File 1), 16 different data sets were generated which were used for independent binary logistic regressions. The p-values from these analyses, listed in Supplementary Table 1B ( Supplementary File 1), were used to pick the most plausible hypothesis *a posteriori*. In each subgroup of data analysis, we find a few hypotheses which had a p value of <0.1 and possibly even <0.05. However, one hypothesis that is significant in the total data analysis is that from case 12 ( Supplementary Table 1B, Supplementary File 1) – that DE in an ordered region with lower charge and higher hydrophobicity context is most relevant for the UPS. Further analysis of the subgroups representing the different pathological conditions suggested a better significance for case 14, but all of them underscored the importance of having DE in the ordered region. In the subgroup comprising the neurodegenerative data set, an ordered region with high hydrophobicity was most relevant.

### Signalling context via phosphorylation of the motif

The DXE motif in the cytoplasmic domain of the membrane proteins has been implicated as a signal, in a different context, for the cargo transport ^25^. It raises questions on whether the same motif could be relevant in other proteins too, especially because DXE has been implicated in the cargo concentration during the protein export from ER. There are several occurrences of DXE or DXXE in our curated data, both in the secretory and non-secretory proteins. Since the unconventional protein secretion is not always active, and it is triggered by cellular stress, we examined the possibility that the there is a phosphorylatable amino acid that might act as an on/off signal in the immediate proximity of DE motif. We screened the curated sequences for a motif XDE, DXE, DEX, XED, EXD, EDX where X is one of the following six amino acids that have the propensity for phosphorylation :- three O-phosphorylated amino acids (S, T, Y) or three N-phosphorylated amino acids (H,R,K) ^26, 27^. The analysis separated the proteins into those which have at least one such motif, and those which do not and studying their relevance for secretion. The contingency tables for the possibility of secretion along with insertions DXE ( Supplementary Table 3, Supplementary File 1) or a general proximity of X ( Supplementary Table 4, Supplementary File 1) do not suggest any significant conclusions, either for the overall data or for the subgroups considered.

### Signalling context via LC3 interacting region (LIR) analysis

The LIR is a motif that aids in autophagy ^28^. We observed that most of the proteins, whether secretory or non-secretory, in our curated data had an LIR motif and thus the proximity of LIR to the DE motif was used for differentiating the different proteins. We explored the hypothesis that DE motif in the immediate proximity of LIR motif (joined in the primary sequence) triggers UPS. Most secretory proteins had DE and LIR motifs that are discontiguous (Dataset 1 ^20^). The group I had 4/5 , group II with 20/26, group IV – secretory proteins within 7/8 and HSPs with 10/11 discontiguous stretches. No significant conclusion could be derived either about the presence of LIR or about its proximity to the DE motif.

### Co-evolutionary context

By using over 3000 homologous sequences (see Methods), pairwise co-evolutionary relationships were constructed among all the amino acids in SOD1 and Acb1. Both these proteins had two DE motifs each, specifically one motif being implicated in UPS (amino acids 77–78 in SOD1 and 23–24 in Acb1) ^16^. We explored to see if the motifs implicated in UPS signalling have co-evolutionary patterns that are conspicuously different from the other two. D102 in SOD1 had a co-evolutionary relation with three other amino acids (amino acids 58,72,81), but a similar pattern could not be seen in the other DE motifs from SOD1 and Acb1. The identification of the DE signature ^16^ involved the knowledge of sequences as well an additional information on the protein localization. Thus sequence alignment and coevolution information alone used in the present analysis could not distinguish the DE that acts as a UPS signature.

## Discussion

The role of diacidic motif DE as a component of retention or export signals has been noted in several contexts ^29^. For example, it had long been known that KDEL or HDEL sequences at the C-terminus act as retention signal to keep the proteins in ER ^29^. Further, DXE was identified as the signal for the export of transmembrane proteins such as VSV-G by COPII-coated vesicles from the endoplasmic reticulum to Golgi ^25^. Although exporting proteins containing these signals work through conventional secretory pathway, DE signal in SOD1 directs the protein export through the UPS. Thus, it is clear that the same DE motif depending on its flanking residues and insertions can assume a different signalling role ^16^. In this work, we examine several eukaryotic proteins to find clues for the patterns leading to UPS.

Since the knowledge of the factors contributing to unconventional secretion is in its nascent stages, the aim of this work was not to predict the possibility of cargo secretion by unconventional pathways but rather to build a hypothesis which may be accepted or rejected as and when more data will be available. The known secreted cargoes that take the UPS route are limited. In this direction, we curated available data which could allow us to make comparisons between the secretory and non-secretory proteins. Towards this, we gathered data from different pathological conditions – cancer secretome, neurodegeneration, and cellular stress. In terms of analysis, we examined the occurrence of DE motif in four different contexts, namely in ordered regions such as helix or sheets, in disordered regions such as loops, or alternatively in the border region between the ordered and disordered regions. We also considered the possible post-translational modifications of the “X” in the DXE motif, co-evolution of DE motif in Acb1 and SOD1, and charge, hydrophobicity and order of DE motif. Specifically, the physical context defining DE was further dissected into 16 different cases to build a hypothesis to identify the most relevant DE among all that were present in the proteins analyzed.

Our analyses suggest that when the diacidic motif DE appears in an ordered structural environment with lower charge and higher hydrophobicity, it is likely to increase the odds of unconventional protein secretion. While the overall *p*-values for the binary logistic regression predictions were significant in some of the cases, the dependence on some of the individual variables used was not significant. One of the reasons for this observation might be the limited sample size of UPS cargoes. There are two ways to validate the hypothesis. The first is to curate data on more eukaryotic proteins which certainly go through UPS and ascertain if the statistics improve significantly. Alternatively, since the hypothesis is centred around the structural order and charge on the amino acids flanking DE, SOD1 secretion may be re-examined with mutations which promotes structural order or by increasing the charges, in addition to the substitutions to alanine that have been already explored by the Malhotra group ^16^.

Considering the alternative hypothesis proposed in this work that DXE motif could be a signatory sequence for UPS, it is plausible that the post-translational modifications, especially, phosphorylation of the inserted amino acids may act as a signal for UPS. It was observed that among the analysed data, the amino acids with propensity for hydroxyl or amino group phosphorylation (O- or N-phosphorylation) are inserted as DXE in the proteins that undergo UPS. Contrary to this, the DXE motif that was seen in COPII is with aliphatic amino acid insertions ^25^. Serine, threonine, tyrosine, as well as arginine ^30^, histidine and lysine ^28^ can undergo phosphorylation under oxidative stress, which is also known to be the external cue that triggers UPS. This hypothesis can be validated by searching for similar patterns in a larger dataset as well as by introducing the DXE motif and observing its chances for unconventional protein secretion.

From the binary logistic regression and odds ratio analysis of the different groups ( Figure 2 and Supplementary Table 2, Supplementary File 1), it is evident that the secretion may be favoured by the microenvironment inside and outside the cell in the pathophysiological conditions. For example, altered proteome ^19^, intracellular pH ^31^, surface charge of the tumour cells ^32^, etc., may allow proteins with buried DXE motif to be exposed and accessible for the UPS machinery as has been suggested for the secretion of Acb1 and SOD1 ^16^. Our analysis that the odds of secretion is significant in terms of minimum charge and maximum hydrophobicity ( Supplementary Table 2 and Supplementary Table 5, Supplementary File 1) is in agreement with the propensity of UPS cargoes to be efficiently secreted in an altered microenvironment, as seen in cancer and neurodegenerative diseases ^33^ such as Parkinson’s disease, Alzheimer’s disease, Huntington disease and ALS.

We also tested the hypothesis that DE motif in the immediate proximity of LIR motif (joined in the primary sequence) triggers UPS. Most secretory proteins had DE and LIR motifs that are discontiguous (Dataset 1 ^20^). We did not observe any significance in the proximal position of the LIR motifs with the DE signal. Moreover, it is seen that the sequence alignment and coevolution information alone used in the present analysis could not distinguish the DE that acts as a UPS signature.

## Conclusions

In this work, we introduce a hypothesis that the diacidic motif DE, when present in the appropriate context, which may be either structural, evolutionary, or aiding in signalling, acts as a unconventional secretion. Despite the limited availability of data, we find the conditions for the odds of unconventional secretion to be significant. The hypothesis about structural order flanking DE or about the phosphorylation of the insertion DXE or the relevance of the LIR motif that is proximal to the DE motif can be validated either with larger data sets, and/or may be a cue for experimental validation. With this study, we hope to raise testable hypotheses about the recognition of proteins secreted by unconventional pathways, something which remains underexplored as yet.

## Data availability

Data underlying these results is available from Open Science Framework.

OSF. Dataset 1: UPS Cargos https://doi.org/10.17605/OSF.IO/2DMT4 ^20^

Data is available under a CC0 1.0 Universal license

The PDB ID and the Uniprot ID of all the proteins used in this study were retrieved from https://www.rcsb.org/ and https://www.uniprot.org/, and are listed in Dataset 1 ^20^.

## Supplementary material

Supplementary File 1: Word document containing the following supplementary material-

**Supplementary Table 1**. **(A)** All possible cases considered for analysis. If at least one DE motif is present in disordered region (or ordered region) then disordered (or ordered) is important. There are cases where the DE motif is in the border between ordered and disordered region. We have looked those as both ordered (O) and disordered (D) cases. **(B)** Probability of various cases considering the three factors (Order, charge and hydrophobicity) affecting the secretion process and the p-value for each case with individual p-value for each variable.

**Supplementary Table 2**. Binary logistic regression –odds ratio of the groups analysed

**Supplementary Table 3: Propensity of the amino acid insertion at DXE sites among secretory and non-secretory proteins**: Among the secretory and non-secretory proteins with a DXE motif, we analyzed to see how often X is one of the three O-phoshporylated amino acids (S, T, Y) or one of the three N-phosphorylated amino acids (H, R, K).

**Supplementary Table 4: Propensity of the amino acid insertion at DEX/EDX sites among secretory and non-secretory proteins**: Among the secretory and non-secretory proteins with a DEX/EDX motif, we analyzed to see how often X is one of the three O-phoshporylated amino acids (S, T, Y) or one of the three N-phosphorylated amino acids (H, R, K).

**Supplementary Table 5:** Secondary structure of DE motif in secretory and non-secretory proteins. The DE motif is ordered (O) if they are in α-helix or β-sheets and disordered (D) if they are in loop or missing residues. Separate cases has been considered if they are in between ordered and disordered.

**Supplementary Figure 1: Comparison between the secretory and non-secretory proteins with their charges and hydrophobicity for various groups.**
