# Supplementary material for: Exploring the context of diacidic motif DE as a signal for unconventional protein secretion in eukaryotic proteins
Source: Wellcome Open Res. 2018 Nov 19;3:148. [Version 1] doi: 10.12688/wellcomeopenres.14914.1 (PMC6305234; doi:10.12688/wellcomeopenres.14914.1)

**Exploring the context of diacidic motif DE as a signal for unconventional protein secretion in eukaryotic proteins**

Sreedevi Padmanabhan^§^, Malay Ranjan Biswal^¶^, Ravi Manjithaya^§^, Meher K. Prakash^¶^*

^§^Autophagy Laboratory, Molecular Biology and Genetics Unit

^¶^Computational Biophysics Group, Theoretical Sciences Unit

Jawaharlal Nehru Centre for Advanced Scientific Research

Jakkur PO, Bangalore, 560064

INDIA

* Corresponding author

-------

**Supplementary Information**

**Supplementary Table 1A**. All possible cases considered for analysis. If at least one DE motif is present in disordered region (or ordered region) then disordered (or ordered) is important. There are cases where the DE motif is in the border between ordered and disordered region. We have looked those as both ordered (O) and disordered (D) cases.

| **CASE** | **Important** | **Bordered region in D / O** | **Parameters sorted for analysis** |
| --- | --- | --- | --- |
| 1 | Disordered is important | Border as D | min C (max H) |
| 2 | Disordered is important | Border as D | max C (min H) |
| 3 | Disordered is important | Border as D | min H (max C) |
| 4 | Disordered is important | Border as D | max H (min C) |
| 5 | Disordered is important | Border as O | min C (max H) |
| 6 | Disordered is important | Border as O | max C (min H) |
| 7 | Disordered is important | Border as O | min H (max C) |
| 8 | Disordered is important | Border as O | max H (min C) |
| 9 | Ordered is important | Border as D | min C (max H) |
| 10 | Ordered is important | Border as D | max C (min H) |
| 11 | Ordered is important | Border as D | min H (max C) |
| 12 | Ordered is important | Border as D | max H (min C) |
| 13 | Ordered is important | Border as O | min C (max H) |
| 14 | Ordered is important | Border as O | max C (min H) |
| 15 | Ordered is important | Border as O | min H (max C) |
| 16 | Ordered is important | Border as O | max H (min C) |

**Supplementary Table 1B**. Probability of various cases considering the three factors (Order, charge and hydrophobicity) affecting the secretion process and the p-value for each case with individual p-value for each variable.

|  | **CASE** | **NN** | **NS** | **SN** | **SS** | **P VALUE** | **P VALUE Charge** | **P VALUE Hydro-phobicity** | **P VALUE Ordered or disordered** | **ODDS RATIO** |
| --- | --- | --- | --- | --- | --- | --- | --- | --- | --- | --- |
| Total data | 1 | 5 | 5 | 34 | 45 | 0.743 | 0.52335077 | 0.74117067 | 0.45990978 | 1.3235 |
|  | 2 | 10 | 8 | 29 | 42 | 0.2963 | 0.53547361 | 0.24467759 | 0.45990978 | 1.8103 |
|  | 3 | 15 | 10 | 24 | 40 | 0.062 | 0.20170712 | 0.47910501 | 0.45990978 | 2.5000 |
|  | 4 | 5 | 4 | 34 | 46 | 0.4974 | 0.94828779 | 0.94354872 | 0.45990978 | 1.6912 |
|  | 5 | 4 | 8 | 35 | 42 | 0.5393 | 0.66226096 | 0.25258818 | 0.6800891 | 0.6000 |
|  | 6 | 12 | 8 | 27 | 42 | 0.1266 | 0.37024255 | 0.35584703 | 0.6800891 | 2.3333 |
|  | 7 | 13 | 9 | 26 | 41 | 0.1371 | 0.14390585 | 0.38709828 | 0.6800891 | 2.2778 |
|  | 8 | 13 | 6 | 26 | 44 | 0.0195 | 0.91155038 | 0.12942052 | 0.6800891 | 3.6667 |
|  | 9 | 17 | 14 | 22 | 36 | 0.1784 | 0.96939488 | 0.93511988 | 0.10296974 | 1.9870 |
|  | 10 | 19 | 14 | 20 | 36 | 0.0504 | 0.8178951 | 0.91411627 | 0.10296974 | 2.4429 |
|  | 11 | 16 | 14 | 23 | 36 | 0.2593 | 0.52518328 | 0.81263204 | 0.10296974 | 1.7888 |
|  | 12 | 19 | 11 | 20 | 39 | 0.0125 | 0.87029653 | 0.4019172 | 0.10296974 | 3.3682 |
|  | 13 | 15 | 10 | 24 | 40 | 0.062 | 0.76985345 | 0.85811818 | 0.09214093 | 2.5000 |
|  | 14 | 15 | 11 | 24 | 39 | 0.1048 | 0.80155333 | 0.75725733 | 0.09214093 | 2.2159 |
|  | 15 | 15 | 10 | 24 | 40 | 0.062 | 0.95850822 | 0.90426609 | 0.09214093 | 2.5000 |
|  | 16 | 15 | 11 | 24 | 39 | 0.1048 | 0.74801161 | 0.66586655 | 0.09214093 | 2.2159 |

|  | **CASE** | **NN** | **NS** | **SN** | **SS** | **P VALUE** | **P VALUE Charge** | **P VALUE Hydro-phobicity** | **P VALUE Ordered or disordered** | **ODDS RATIO** |
| --- | --- | --- | --- | --- | --- | --- | --- | --- | --- | --- |
| Cancer data | 1 | 4 | 4 | 5 | 6 | 1 | 0.67095853 | 0.61483704 | 0.3574488 | 1.2000 |
|  | 2 | 7 | 3 | 2 | 7 | 0.0698 | 0.10232889 | 0.43956727 | 0.3574488 | 8.1667 |
|  | 3 | 6 | 2 | 3 | 8 | 0.0698 | 0.07433041 | 0.90773576 | 0.3574488 | 8.0000 |
|  | 4 | 5 | 4 | 4 | 6 | 0.6563 | 0.76756689 | 0.60830131 | 0.3574488 | 1.8750 |
|  | 5 | 4 | 5 | 5 | 5 | 1 | 0.67095853 | 0.49394376 | 0.3574488 | 0.8000 |
|  | 6 | 7 | 3 | 2 | 7 | 0.0698 | 0.10232889 | 0.43956727 | 0.3574488 | 8.1667 |
|  | 7 | 6 | 2 | 3 | 8 | 0.0698 | 0.07433041 | 0.91650218 | 0.3574488 | 8.0000 |
|  | 8 | 5 | 3 | 4 | 7 | 0.3698 | 0.67607709 | 0.25281586 | 0.3574488 | 2.9167 |
|  | 9 | 7 | 4 | 2 | 6 | 0.1698 | 0.63547255 | 0.15702494 | 0.52483622 | 5.2500 |
|  | 10 | 7 | 2 | 2 | 8 | 0.023 | 0.36951029 | 0.09317746 | 0.52483622 | 14.0000 |
|  | 11 | 5 | 4 | 4 | 6 | 0.6563 | 0.41501484 | 0.16739353 | 0.52483622 | 1.8750 |
|  | 12 | 7 | 3 | 2 | 7 | 0.0698 | 0.49226435 | 0.02125843 | 0.52483622 | 8.1667 |
|  | 13 | 6 | 3 | 3 | 7 | 0.1789 | 0.89814676 | 0.38461264 | 0.85502651 | 4.6667 |
|  | 14 | 7 | 2 | 2 | 8 | 0.023 | 0.14627084 | 0.11574058 | 0.85502651 | 14.0000 |
|  | 15 | 7 | 3 | 2 | 7 | 0.0698 | 0.79608005 | 0.4372128 | 0.85502651 | 8.1667 |
|  | 16 | 6 | 2 | 3 | 8 | 0.0698 | 0.29560122 | 0.03576384 | 0.85502651 | 8.0000 |

|  | **CASE** | **NN** | **NS** | **SN** | **SS** | **P VALUE** | **P VALUE Charge** | **P VALUE Hydro-phobicity** | **P VALUE Ordered or disordered** | **ODDS RATIO** |
| --- | --- | --- | --- | --- | --- | --- | --- | --- | --- | --- |
| Neuro degen-erative and known UPS vs Non secre-tive | 1 | 11 | 9 | 19 | 20 | 0.7847 | 0.67366194 | 0.90573796 | 0.253476 | 1.2865 |
|  | 2 | 11 | 6 | 19 | 23 | 0.2516 | 0.76671223 | 0.69396204 | 0.253476 | 2.2193 |
|  | 3 | 15 | 10 | 15 | 19 | 0.2949 | 0.59505645 | 0.98596278 | 0.253476 | 1.9000 |
|  | 4 | 12 | 9 | 18 | 20 | 0.5889 | 0.66237985 | 0.95494611 | 0.253476 | 1.4815 |
|  | 5 | 18 | 13 | 12 | 16 | 0.3015 | 0.94430682 | 0.46838828 | 0.48680369 | 1.8462 |
|  | 6 | 15 | 11 | 15 | 18 | 0.4348 | 0.85504976 | 0.62077149 | 0.48680369 | 1.6364 |
|  | 7 | 13 | 12 | 17 | 17 | 1 | 0.59351107 | 0.84545674 | 0.48680369 | 1.0833 |
|  | 8 | 16 | 12 | 14 | 17 | 0.4379 | 0.88748041 | 0.19786875 | 0.48680369 | 1.6190 |
|  | 9 | 16 | 9 | 14 | 20 | 0.1154 | 0.57073547 | 0.46249542 | 0.08579865 | 2.5397 |
|  | 10 | 19 | 11 | 11 | 18 | 0.0698 | 0.68415137 | 0.138706 | 0.08579865 | 2.8264 |
|  | 11 | 21 | 10 | 9 | 19 | 0.0092 | 0.88260184 | 0.09029988 | 0.08579865 | 4.4333 |
|  | 12 | 20 | 10 | 10 | 19 | 0.0194 | 0.11286277 | 0.48080154 | 0.08579865 | 3.8000 |
|  | 13 | 14 | 11 | 16 | 18 | 0.601 | 0.3080496 | 0.20242188 | 0.18148992 | 1.4318 |
|  | 14 | 20 | 8 | 10 | 21 | 0.004 | 0.2402378 | 0.03341047 | 0.18148992 | 5.2500 |
|  | 15 | 18 | 10 | 12 | 19 | 0.0692 | 0.83108213 | 0.10846807 | 0.18148992 | 2.8500 |
|  | 16 | 20 | 5 | 10 | 24 | 0.0002 | 0.04402958 | 0.03327044 | 0.18148992 | 9.6000 |

|  | **CASE** | **NN** | **NS** | **SN** | **SS** | **P VALUE** | **P VALUE Charge** | **P VALUE Hydro-phobicity** | **P VALUE Ordered or disordered** | **ODDS RATIO** |
| --- | --- | --- | --- | --- | --- | --- | --- | --- | --- | --- |
| HSP vs non secre-tive | 1 | 30 | 11 | 0 | 0 | 1 | 0.53996776 | 0.81803078 | 0.55477603 | INFINITY |
|  | 2 | 29 | 8 | 1 | 3 | 0.0521 | 0.56072049 | 0.04544889 | 0.55477603 | 10.8750 |
|  | 3 | 30 | 6 | 0 | 5 | 0.0006 | 0.99181059 | 0.02776383 | 0.55477603 | INFINITY |
|  | 4 | 30 | 11 | 0 | 0 | 1 | 1 | 0.77876969 | 0.55477603 | INFINITY |
|  | 5 | 30 | 11 | 0 | 0 | 1 | 0.40613903 | 0.26681367 | 0.55477603 | INFINITY |
|  | 6 | 30 | 11 | 0 | 0 | 1 | 0.99428729 | 0.2740773 | 0.55477603 | INFINITY |
|  | 7 | 29 | 7 | 1 | 4 | 0.0138 | 0.53668059 | 0.02858723 | 0.55477603 | 16.5714 |
|  | 8 | 30 | 11 | 0 | 0 | 1 | 0.86564793 | 0.68233718 | 0.55477603 | INFINITY |
|  | 9 | 30 | 10 | 0 | 1 | 0.2638 | 0.81641702 | 0.88520423 | 0.04569734 | INFINITY |
|  | 10 | 27 | 6 | 3 | 5 | 0.0218 | 0.98882726 | 0.11943886 | 0.04569734 | 7.5000 |
|  | 11 | 27 | 9 | 3 | 2 | 0.5977 | 0.71029099 | 0.31076057 | 0.04569734 | 2.0000 |
|  | 12 | 27 | 7 | 3 | 4 | 0.0692 | 0.36107661 | 0.2940053 | 0.04569734 | 5.1429 |
|  | 13 | 28 | 7 | 2 | 4 | 0.0351 | 0.0566603 | 0.05397491 | 0.08958272 | 8.0000 |
|  | 14 | 27 | 6 | 3 | 5 | 0.0218 | 0.3251823 | 0.09845206 | 0.08958272 | 7.5000 |
|  | 15 | 27 | 8 | 3 | 3 | 0.3162 | 0.95439653 | 0.06102705 | 0.08958272 | 3.3750 |
|  | 16 | 30 | 11 | 0 | 0 | 1 | 0.60727826 | 0.99507312 | 0.08958272 | INFINITY |

|  | **CASE** | **NN** | **NS** | **SN** | **SS** | **P VALUE** | **P VALUE Charge** | **P VALUE Hydro-phobicity** | **P VALUE Ordered or disordered** | **ODDS RATIO** |
| --- | --- | --- | --- | --- | --- | --- | --- | --- | --- | --- |
| HSP and cancer data from group 3A, 3B, and 3C | 1 | 0 | 0 | 9 | 21 | 1 | 0.92517165 | 0.95767476 | 0.35509314 | INFINITY |
|  | 2 | 4 | 0 | 5 | 21 | 0.0046 | 0.11690723 | 0.45404011 | 0.35509314 | INFINITY |
|  | 3 | 3 | 0 | 6 | 21 | 0.0207 | 0.14826929 | 0.54719541 | 0.35509314 | INFINITY |
|  | 4 | 0 | 0 | 9 | 21 | 1 | 0.75782391 | 0.70985758 | 0.35509314 | INFINITY |
|  | 5 | 0 | 0 | 9 | 21 | 1 | 0.92517165 | 0.65317991 | 0.35509314 | INFINITY |
|  | 6 | 4 | 1 | 5 | 20 | 0.0195 | 0.07018604 | 0.86155596 | 0.35509314 | 16.0000 |
|  | 7 | 4 | 1 | 5 | 20 | 0.0195 | 0.09320402 | 0.58811518 | 0.35509314 | 16.0000 |
|  | 8 | 0 | 0 | 9 | 21 | 1 | 0.85616524 | 0.75648518 | 0.35509314 | INFINITY |
|  | 9 | 0 | 1 | 9 | 20 | 1 | 0.76868416 | 0.50102815 | 0.75522839 | 0.0000 |
|  | 10 | 5 | 2 | 4 | 19 | 0.0139 | 0.3426805 | 0.03951339 | 0.75522839 | 11.8750 |
|  | 11 | 4 | 2 | 5 | 19 | 0.0492 | 0.76001799 | 0.07406526 | 0.75522839 | 7.6000 |
|  | 12 | 4 | 2 | 5 | 19 | 0.0492 | 0.15446722 | 0.09528097 | 0.75522839 | 7.6000 |
|  | 13 | 2 | 2 | 7 | 19 | 0.5632 | 0.91796977 | 0.38768914 | 0.27394477 | 2.7143 |
|  | 14 | 5 | 1 | 4 | 20 | 0.0046 | 0.29809661 | 0.05532681 | 0.27394477 | 25.0000 |
|  | 15 | 3 | 2 | 6 | 19 | 0.1432 | 0.65661296 | 0.20732339 | 0.27394477 | 4.7500 |
|  | 16 | 4 | 1 | 5 | 20 | 0.0195 | 0.17476524 | 0.20088486 | 0.27394477 | 16.0000 |

**Supplementary Table 2**. Binary logistic regression –odds ratio of the groups analyzed

Total data

|  | B | Sig. | Odds=Exp(B) |
| --- | --- | --- | --- |
| Charge | 0.016 | 0.931 | 1.016 |
| Hydrophobicity | -0.031 | 0.908 | 0.970 |
| Order | -0.808 | 0.105 | 0.446 |

Neuro degenerative and known UPS vs non-secretory

|  | B | Sig. | Odds=Exp(B) |
| --- | --- | --- | --- |
| Charge | 0.636 | 0.037 | 1.889 |
| Hydrophobicity | 1.062 | 0.011 | 2.892 |
| Order | -0.143 | 0.834 | 0.867 |

HSP vs non-secretory

|  | B | Sig. | Odds=Exp(B) |
| --- | --- | --- | --- |
| Charge | -0.098 | 0.790 | 0.907 |
| Hydrophobicity | -0.903 | 0.097 | 0.405 |
| Order | -2.014 | 0.083 | 0.134 |

Cancer data from Groups 3B, 3C

|  | B | Sig. | Odds=Exp(B) |
| --- | --- | --- | --- |
| Charge | 0.011 | 0.984 | 1.011 |
| Hydrophobicity | -4.526 | 0.050 | 0.011 |
| Order | -5.629 | 0.106 | 0.004 |

Cancer secretion data including HSP secretion (Groups 3A, 3B, 3C)

|  | B | Sig. | Odds=Exp(B) |
| --- | --- | --- | --- |
| Charge | -0.232 | 0.568 | 0.793 |
| Hydrophobicity | -2.370 | 0.031 | 0.093 |
| Order | -3.185 | 0.056 | 0.041 |

**Supplementary Table 3:** **Propensity of the amino acid insertion at DXE sites among secretory and non-secretory proteins**: Among the secretory and non-secretory proteins with a DXE motif, we analyzed to see how often X is one of the three O-phoshporylated amino acids (S, T, Y) or one of the three N-phosphorylated amino acids (H, R, K).

Total data

| **D-X-E insertion** | **Insertion X=S,T,Y,H,R,K** | **Other insertions** |
| --- | --- | --- |
| Secreted | 37 | 10 |
| Unsecreted | 28 | 10 |

Neurodegenerative and known UPS vs non-secretory

| **D-X-E insertion** | **Insertion X=S,T,Y,H,R,K** | **Other insertions** |
| --- | --- | --- |
| Secreted | 18 | 9 |
| Unsecreted | 23 | 5 |

Cancer data from Groups 3B, 3C

| **D-X-E insertion** | **Insertion X=S,T,Y,H,R,K** | **Other insertions** |
| --- | --- | --- |
| Secreted | 10 | 0 |
| Unsecreted | 5 | 5 |

HSP vs non-secretory

| **D-X-E insertion** | **Insertion X=S,T,Y,H,R,K** | **Other insertions** |
| --- | --- | --- |
| Secreted | 9 | 1 |
| Unsecreted | 23 | 5 |

Cancer secretion data including HSP secretion (Groups 3A, 3B, 3C)

| **D-X-E insertion** | **Insertion X=S,T,Y,H,R,K** | **Other insertions** |
| --- | --- | --- |
| Secreted | 19 | 1 |
| Unsecreted | 5 | 5 |

- 15 proteins do not have DXE
- Only 85 proteins are considered for calculation.

**Supplementary Table 4:** **Propensity of the amino acid insertion at DEX/EDX sites among secretory and non-secretory proteins**: Among the secretory and non-secretory proteins with a DEX/EDX motif, we analyzed to see how often X is one of the three O-phoshporylated amino acids (S, T, Y) or one of the three N-phosphorylated amino acids (H, R, K).

Total data

| **DEX/EDX insertion** | **Insertion X=S,T,Y,H,R,K** | **Other insertions** |
| --- | --- | --- |
| Secreted | 42 | 8 |
| Unsecreted | 29 | 10 |

Neurodegenerative and known UPS vs non-secretory

| **DEX/EDX insertion** | **Insertion X=S,T,Y,H,R,K** | **Other insertions** |
| --- | --- | --- |
| Secreted | 23 | 6 |
| Unsecreted | 20 | 10 |

Cancer data from Groups 3B, 3C

| **DEX/EDX insertion** | **Insertion X=S,T,Y,H,R,K** | **Other insertions** |
| --- | --- | --- |
| Secreted | 8 | 2 |
| Unsecreted | 9 | 0 |

HSP vs non-secretory

| **DEX/EDX insertion** | **Insertion X=S,T,Y,H,R,K** | **Other insertions** |
| --- | --- | --- |
| Secreted | 11 | 0 |
| Unsecreted | 20 | 10 |

Cancer secretion data including HSP secretion (Groups 3A, 3B, 3C)

| **DEX/EDX insertion** | **Insertion X=S,T,Y,H,R,K** | **Other insertions** |
| --- | --- | --- |
| Secreted | 19 | 2 |
| Unsecreted | 9 | 0 |

- 11 proteins do not have DE
- Only 89 proteins are considered for calculation.

**Supplementary Table 5:** Secondary structure of DE motif in secretory and non-secretory proteins. The DE motif is ordered (O) if they are in α-helix or β-sheets and disordered (D) if they are in loop or missing residues. Separate cases has been considered if they are in between ordered and disordered.

Total data

|  | **Disordered is important &**  **B is Disordered** | |  | **Disordered is important &**  **B is ordered** | |  | **Ordered is important &**  **B is Disordered** | |  | **Ordered is important &**  **B is ordered** | |
| --- | --- | --- | --- | --- | --- | --- | --- | --- | --- | --- | --- |
|  | **O** | **D** |  | **O** | **D** |  | **O** | **D** |  | **O** | **D** |
| S | 4 | 46 |  | 5 | 45 |  | 33 | 17 |  | 39 | 11 |
| N | 5 | 34 |  | 5 | 34 |  | 19 | 20 |  | 24 | 15 |

Neurodegenerative and known UPS vs non-secretory

|  | **O** | **D** |  | **O** | **D** |  | **O** | **D** |  | **O** | **D** |
| --- | --- | --- | --- | --- | --- | --- | --- | --- | --- | --- | --- |
| S | 2 | 27 |  | 3 | 26 |  | 20 | 9 |  | 23 | 6 |
| N | 5 | 25 |  | 5 | 25 |  | 14 | 16 |  | 19 | 11 |

Cancer data from Groups 3B, 3C

|  | **O** | **D** |  | **O** | **D** |  | **O** | **D** |  | **O** | **D** |
| --- | --- | --- | --- | --- | --- | --- | --- | --- | --- | --- | --- |
| S | 1 | 9 |  | 1 | 9 |  | 4 | 6 |  | 6 | 4 |
| N | 0 | 9 |  | 0 | 9 |  | 5 | 4 |  | 5 | 4 |

HSP vs non-secretory

|  | **O** | **D** |  | **O** | **D** |  | **O** | **D** |  | **O** | **D** |
| --- | --- | --- | --- | --- | --- | --- | --- | --- | --- | --- | --- |
| S | 1 | 10 |  | 1 | 10 |  | 9 | 2 |  | 10 | 1 |
| N | 5 | 25 |  | 5 | 25 |  | 14 | 16 |  | 19 | 11 |

Cancer secretion data including HSP secretion (Groups 3A, 3B, 3C)

|  | **O** | **D** |  | **O** | **D** |  | **O** | **D** |  | **O** | **D** |
| --- | --- | --- | --- | --- | --- | --- | --- | --- | --- | --- | --- |
| S | 1 | 10 |  | 1 | 10 |  | 9 | 2 |  | 10 | 1 |
| N | 5 | 25 |  | 5 | 25 |  | 14 | 16 |  | 19 | 11 |

- 11 proteins do not have DE
- Only 89 proteins are considered for calculation.

**Supplementary Figure 1: Comparison between the secretory and non-secretory proteins with their charges and hydrophobicity for various groups.**

Total data


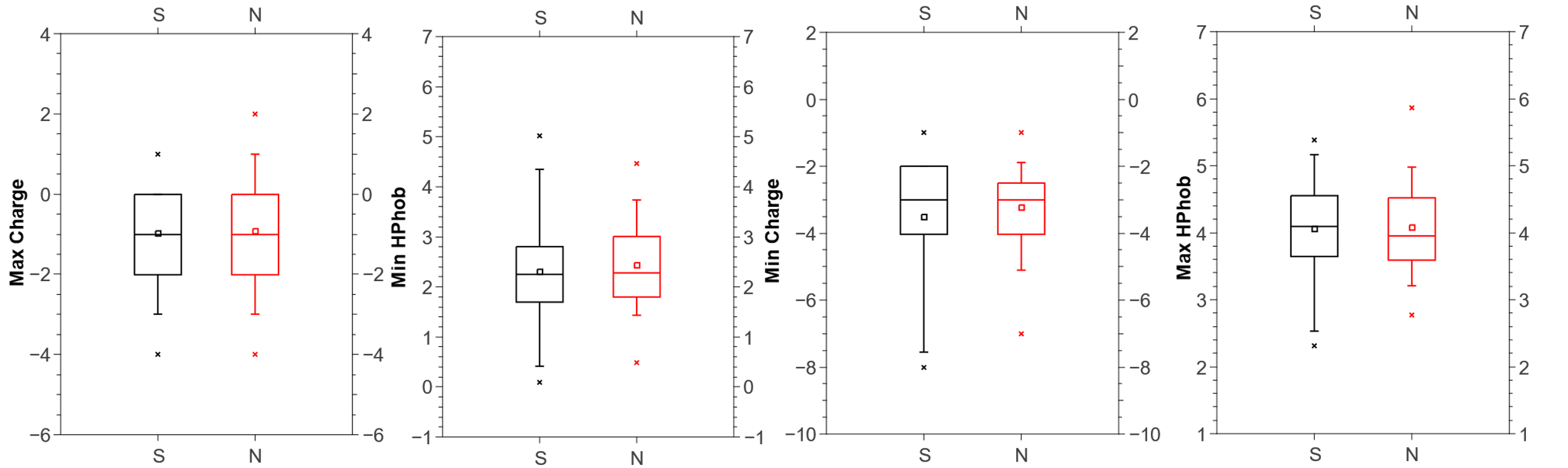


Neurodegenerative and known UPS vs non-secretory


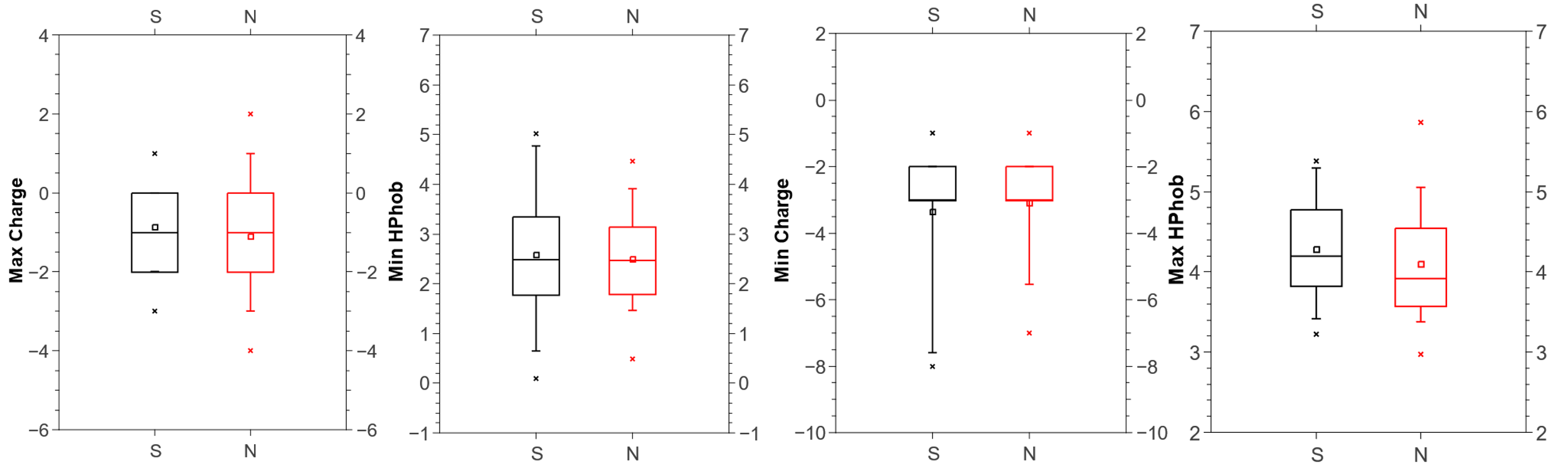


HSP vs non-secretory


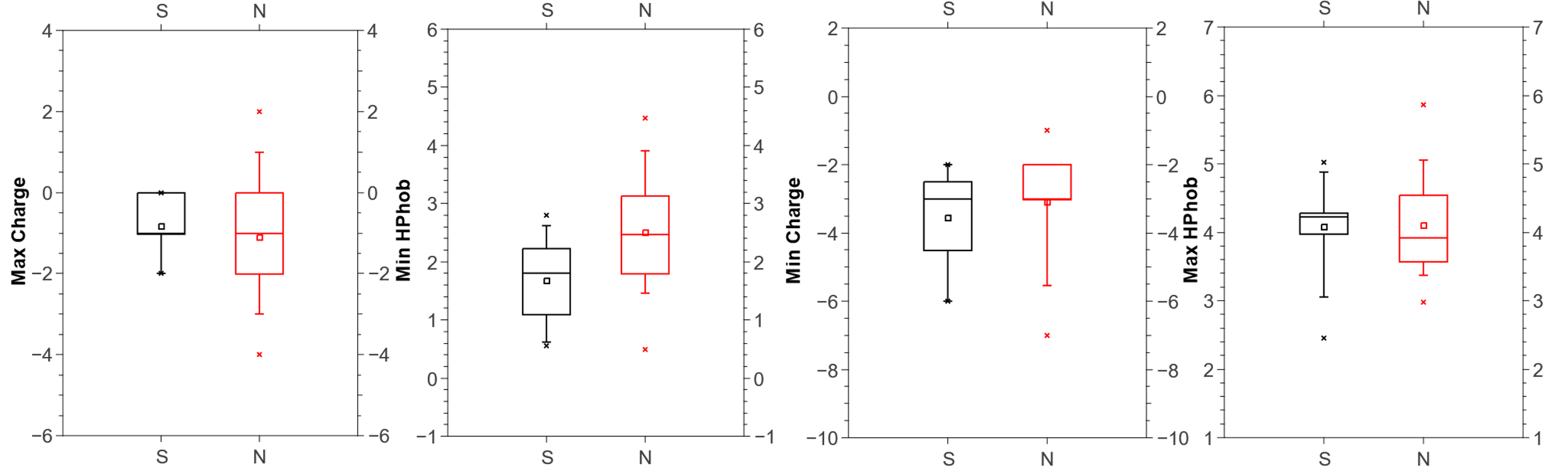


Cancer secretion data including HSP secretion (Groups 3A, 3B, 3C)


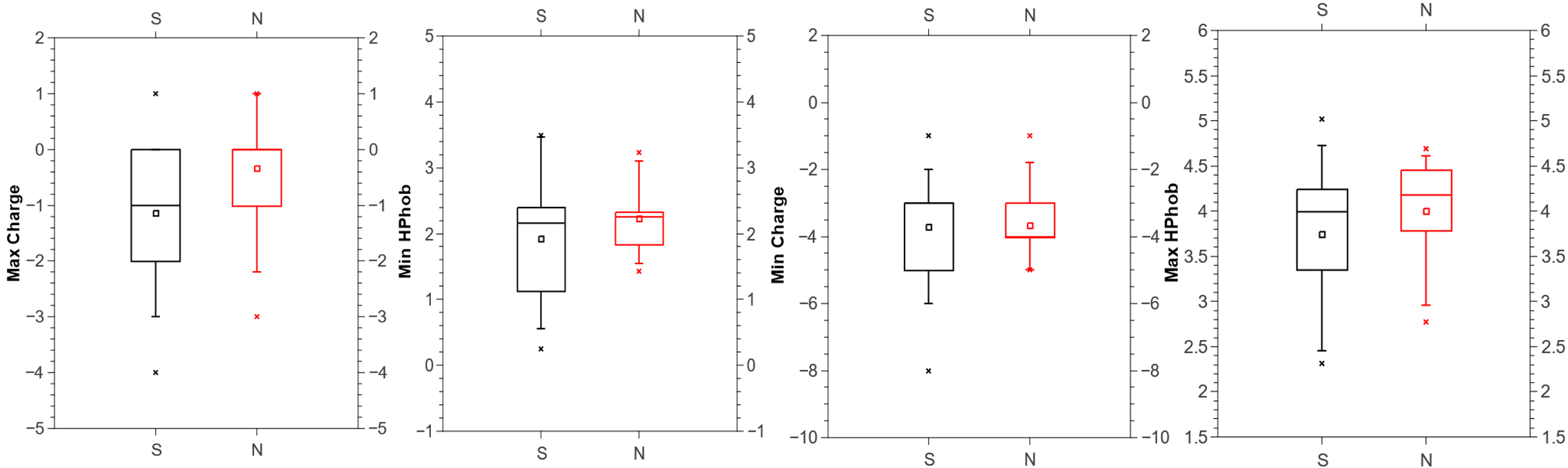

Supplement: Supplementary file 1 [file wellcomeopenres-3-16259-s0000.tgz › 5c08475e-42bb-418b-99dd-62d5421ec9c0_Supplementary_Table_-_2Nov_2018.docx]
