# Supplementary material for: Exploring the context of diacidic motif DE as a signal for unconventional protein secretion in eukaryotic proteins
Source: Wellcome Open Res. 2018 Nov 19;3:148. [Version 1] doi: 10.12688/wellcomeopenres.14914.1 (PMC6305234; doi:10.12688/wellcomeopenres.14914.1)
Supplement: Peer review history

# Peer review: Referee response for version 1

**DOI:** 10.21956/wellcomeopenres.16259.r34305

**Published:** 2018-12-14

**Authors:** Suresh Subramani

**Affiliations:** 1 Division of Biological Sciences, University of California, San Diego, La Jolla, CA, 92093-0322, USA

While proteins secreted by the classical secretory pathway have clearly identifiable signal sequences that target them for insertion across the endoplasmic reticulum membrane, many proteins that lack such canonical sequences and that are secreted by one of several unconventional secretion pathways (UPSs) have not been characterized sufficiently to understand whether they have a common signal for the UPS pathway. These authors address this important question. Work from the Malhotra lab identified such a sequence in one protein, superoxide dismutase (SOD1) from human and mouse cells. This turned out to be a diacidic (DE) motif. However, because such a minimal sequence could be present in many proteins, not all of which are likely to be substrates for UPS, and similar (HDEL, KDEL or DXE sequences)  are used in other sorting pathways, the authors have used a bioinformatic approach to compare 100 different eukaryotic proteins, 57 secretory and 43 non-secretory, with DE sequences. They examined features, such as hydrophobicity, charge and protein order, in the vicinity of the diacidic motif, in proteins sorted via the UPS, but that are absent in non-secretory proteins.. They also looked for the presence of a proximal LIR motif presumably because the UPS relies on autophagy. The authors suggest that suggest that when the diacidic motif DE appears in an ordered structural environment with lower charge and higher hydrophobicity, it is likely to increase the odds of unconventional protein secretion. They did not find a significant presence of a proximal LIR.

 The paper presents reasonable and testable hypotheses, but falls short in leaving the testing to others, or for the accumulation of a larger list of UPS cargos. It seems reasonable to publish this as a hypothesis.

I have read this submission. I believe that I have an appropriate level of expertise to confirm that it is of an acceptable scientific standard.

# Peer review: Referee response for version 1

**DOI:** 10.21956/wellcomeopenres.16259.r34309

**Published:** 2018-12-04

**Authors:** Vivek Malhotra

**Affiliations:** 1 Centre for Genomic Regulation, The Barcelona Institute of Science and Technology, Barcelona, Spain

The DE motif has been identified in proteins like SOD1 and Acb1 for their ER-Golgi independent pathway of secretion. But this di-acidic motif is quite common and it is unclear how cells exploit this signal to export a specific class of proteins. Prakash and colleagues provide useful clues to help place the significance of these residues as a potential export signal. They find that it helps if DE sequence  is contained in the ordered domains of the proteins with less charge and higher overall hydrophobicity. This could in principle be used for membrane attachment. They also suggest the significance of LIR sequences in close proximity to increase the potential usage of the di-acidic motif for export. This is particularly interesting because LIR containing proteins have specific membrane associated receptors and the cells use this combination to clear cellular proteins by autophagy.  A combination of DE and LIR motif could be used to bind specific membranes for export and to  avoid autophagic clearance. This information will help move the field forward and I strongly support the publication of this data.

I have read this submission. I believe that I have an appropriate level of expertise to confirm that it is of an acceptable scientific standard.
